# Temperature Characteristics of a Contour Mode MEMS AlN Piezoelectric Ring Resonator on SOI Substrate

**DOI:** 10.3390/mi12020143

**Published:** 2021-01-29

**Authors:** Sitao Fei, Hao Ren

**Affiliations:** 1 School of Information Science and Technology, ShanghaiTech University, Shanghai 201210, China; feist@shanghaitech.edu.cn; 2 Shanghai Institute of Microsystem and Information Technology, Chinese Academy of Sciences, Shanghai 200050, China; 3 University of Chinese Academy of Sciences, Beijing 100049, China

**Keywords:** MEMS AlN resonator, contour mode, heavily doped silicon, temperature coefficient of frequency (TCF), cryogenic characteristics

## Abstract

As a result of their IC compatibility, high acoustic velocity, and high thermal conductivity, aluminum nitride (AlN) resonators have been studied extensively over the past two decades, and widely implemented for radio frequency (RF) and sensing applications. However, the temperature coefficient of frequency (TCF) of AlN is −25 ppm/°C, which is high and limits its RF and sensing application. In contrast, the TCF of heavily doped silicon is significantly lower than the TCF of AlN. As a result, this study uses an AlN contour mode ring type resonator with heavily doped silicon as its bottom electrode in order to reduce the TCF of an AlN resonator. A simple microfabrication process based on Silicon-on-Insulator (SOI) is presented. A thickness ratio of 20:1 was chosen for the silicon bottom electrode to the AlN layer in order to make the TCF of the resonator mainly dependent upon heavily doped silicon. A cryogenic cooling test down to 77 K and heating test up to 400 K showed that the resonant frequency of the AlN resonator changed linearly with temperature change; the TCF was shown to be −9.1 ppm/°C. The temperature hysteresis characteristic of the resonator was also measured, and the AlN resonator showed excellent temperature stability. The quality factor versus temperature characteristic was also studied between 77 K and 400 K. It was found that lower temperature resulted in a higher quality factor, and the quality factor increased by 56.43%, from 1291.4 at 300 K to 2020.2 at 77 K.

## 1. Introduction

Nowadays, microelectromechanical systems (MEMS) are being studied extensively because of their small footprint, low cost, batch fabrication, and microelectronic fabrication compatibility. A variety of applications have been presented that benefit from the advantages of MEMS, such as accelerometers, gyroscopes, micromirrors, actuators, fuel cells, biosensors, resonators, etc. [1,2,3,4,5,6,7,8]. Piezoelectric MEMS resonators, as one of the most important MEMS topics, have been widely studied due to their high frequency, high quality factor, small size, low phase noise, low power consumption, and low cost. Since the invention of resonators, quartz crystal resonators have played an important role in consumer, commercial, industrial, and military products [9]. Because of their high quality and good temperature stability, the market for quartz crystal resonators is strong. However, they are poorly suited to monolithic integration onto silicon wafer, which means that they are not compatible with IC microfabrication. As a result, the costs cannot be significantly reduced [10]. MEMS resonators that are compatible with IC fabrication may be a good solution to reducing the costs.

MEMS resonators can be divided into two categories based on operating principle: one implements electrostatic methods, such as capacitive resonators, and the other implements piezoelectric methods via the use of a piezoelectric material [11]. For piezoelectric resonators, four kinds of piezoelectric materials are commonly implemented: lead zirconate titanate (PZT), zinc oxide (ZnO), lithium niobate (LiNbO_3_), and AlN. PZT is one of the most widely used piezoelectric materials, LiNbO_3_ has a high electromechanical coupling coefficient, and ZnO and AlN each have significant advantages in terms of their compatibility with IC technology [12,13,14]. Among these four materials, AlN has the highest acoustic wave velocity and excellent thermal conductivity [15,16]. Therefore, various AlN-based resonators have been reported, such as flexural mode resonators [17], film bulk acoustic wave resonators (FBAR) [18], contour mode resonators [19], and lamb wave resonators [20]. Different modes have different frequency ranges; for contour mode, the main frequency is around 10 MHz to 10 GHz, while that of flexural mode is often smaller than 10 MHz. Among the different modes, contour mode resonators have an advantage, in that their resonant frequency is mainly determined by their lateral dimensions, which means that their center frequency is set primarily by a lithographic process; therefore, it is easy to operate at several different frequencies on the same chip [21].

As a result of the rapid development of wireless communication systems from 2G to 4G and 5G, the requirements for RF carrier frequencies have increased, which makes AlN resonators attractive due to their high operation frequency. In addition, the resonant frequency over temperature is also important for resonators. The temperature coefficient of frequency (TCF) of resonators needs to be small for applications such as filters [22] and sensors [23]. However, the TCF of AlN is −25 ppm/°C [24], which makes it difficult to be implemented in filters and sensors [25]. Unlike temperature-compensated crystal oscillators (TCXOs) based on AT-cut quartz, which offers outstanding performance and long-term temperature stability [26], MEMS resonators generally require extra temperature compensation techniques.

To overcome the obstacle of high TCF, various approaches have been adopted to compensate for temperature variations, which can be divided into active and passive temperature compensation technologies [27]. Active compensation technologies, such as electronic phase-locked loop (PLL) and oven storage, have been widely applied. For instance, Salvia et al. achieved ±0.05 ppm by PLL technology and Kwon et al. achieved ±1.5 ppb by oven storage around the 100 °C range [28,29]. The drawbacks of an active approach are the inevitable circuit complexity and power consumption caused by additional sensors. In passive compensation technologies, composite structures, such as silicon oxide as an additional layer under the piezoelectric material, have been widely adopted. The positive TCF of such structures can compensate for the negative TCF of AlN by choosing an appropriate thickness ratio [30]. The geometric stress compensation method utilizing a mechanical support structure can also reduce TCF by introducing stress to counteract temperature-induced frequency shifts [31,32]. Although the passive temperature compensation method does not complicate the circuit or increase power consumption, the drawback is increasing the fabrication complexity.

In recent years, another method applying degenerated doping silicon to compensate for temperature variation has been presented. The TCF of silicon is strongly influenced by its doping concentration, and the TCF can be reduced from −30 ppm/°C (native silicon) to −8 ppm/°C (highly doped silicon) [33]. Applying this technology, a thickness-lamé mode resonator was designed. However, the reported temperature range was −20 to 180 °C, and the TCF varied with temperature [34]. The mode shapes of heavily doped silicon resonator also affected the TCF. Length extensional mode, width extensional mode, lamé mode, square extensional mode, and ring mode were studied in [35], and the ring mode shape displayed a smaller TCF compared to other modes in n-type highly doped silicon. In this study, a contour mode ring type AlN resonator with n-type heavily doped silicon is presented. The resonant frequency is 144.03 MHz, and the electromechanical coupling coefficient is 0.198%. Based on the results of previous studies [36], this paper mainly focuses on the temperature characteristics of the contour mode AlN resonator. A constant TCF of 9.1 ppm/°C is demonstrated in the measurement temperature range of 77 K to 400 K. The total structure is simply fabricated on a Silicon-on-Insulator (SOI) substrate. Not only simplifying the fabrication complexity, the thickness ratio of silicon bottom electrode to AlN layer is 20:1, which means that the TCF is mainly determined by silicon. The TCF of heavily doped silicon is significantly reduced, which reduces the TCF of the AlN resonator. In the next section, a design model and theoretical analysis are presented. Section 3 is the simple fabrication process of the AlN resonator, based on n-type heavily doped silicon. Section 4 displays the device characterization, including surface roughness, crystallographic structure, and admittance versus frequency measurement. The cryogenic cooling and heating measurement ranges from 77 K to 400 K are applied to test the TCF. Lastly, the conclusion is presented in Section 5.

## 2. Device Design and Modelling

**Figure 1:**
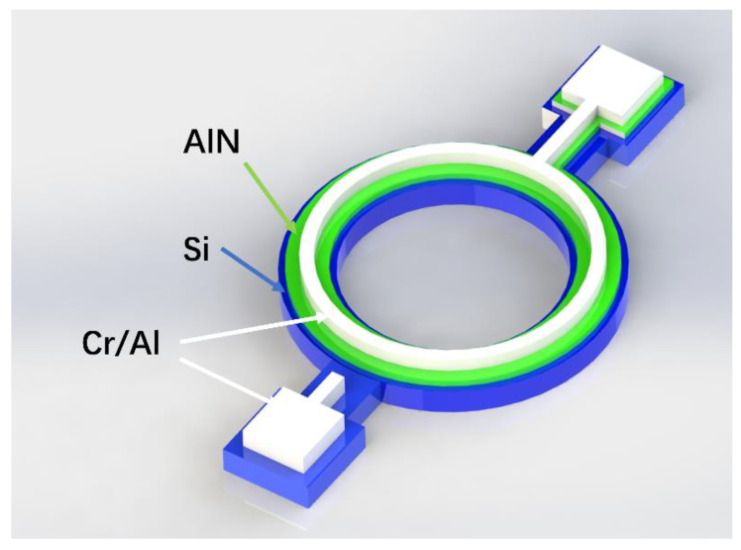
Schematic of the contour mode ring type resonator.

Figure 1 shows the physical structure of the resonator. The three colored layers denote different materials. The blue layer at the bottom is the silicon wafer as the bottom electrode, the green layer is the piezoelectric part using AlN, and the top white layer is the top electrode made of Cr/Al. Traditionally, the electrode material used is either Pt or Mo and Al has the tendencies of surface oxidation [37]. However, the deposition thickness of Al can be 1 μm, which reduces the resistance of the Al interconnect, and the Young’s modulus of Al (70 GPa) is smaller than Pt and Mo (168 GPa and 312 GPa), which has less of an effect on the TCF of the resonator when their thicknesses are the same. The AlN layer was sandwiched between two electrodes, making the electric field cover the piezoelectric material to maximize the vibration. The whole structure was based on a simple microfabrication process on a SOI substrate, which can reduce the fabrication complexity.

**Figure 2:**
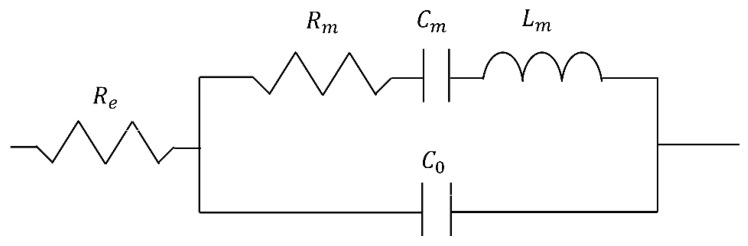
Lumped-element of the Butterworth Van Dyke (BVD) model of the aluminum nitride (AlN) resonator.

The BVD (Butterworth Van Dyke) model is a simple physical model for acoustic resonator modeling that was applied in this study to characterize the one-port lamb wave resonator [38]. The model of a lumped-element equivalent circuit is shown in Figure 2. *C*_0_ is the static parallel capacitance, *R*_m_ is the motional resistance, *C*_m_ is the motional capacitance, *L*_m_ is the motional inductance, and *R*_e_ is the series resistance of electrode. *C*_0_, *R*_m_, *C*_m_, *L*_m_, and *f*_s_ are defined as [39].

(1) $$C_{0}=\varepsilon_{0}\varepsilon_{33}\frac{2\pi r_{\mathrm{ave}}w}{t}$$

(2) Rm=π28t2πraveρ12EP32Qd312

(3) $$C_{m}=\frac{8}{\pi }\frac{2wr_{\mathrm{ave}}}{t}d_{31}^{2}E_{P}$$

(4) $$L_{m}=\frac{\rho }{8}\frac{wt}{2\pi r_{\mathrm{ave}}}\frac{1}{d_{31}^{2}E_{P}^{2}}$$

(5) $$f_{s}=\frac{1}{2\pi \sqrt{C_{m}L_{m}}}=\frac{1}{2w}\sqrt{\frac{E_{P}}{\rho }}$$

where $\varepsilon_{33}$ = 9 is the relative dielectric constant of AlN, t=0.5 μm is the thickness, $\rho =3300\;\mathrm{kg}/m^{3}$ is the density of the AlN film, w=13.5 μm is the ring width, $r_{\mathrm{ave}}=160\;\mu m$ is the average radius of the ring, $d_{31}=-1.9159\times 10^{-12}\;C/N$ is the piezoelectric coefficient, $E_{P}=0.99\times 10^{11\;}\mathrm{Pa}$ is the equivalent Young’s modulus of AlN, *Q* is the quality factor, and *f*_s_ is the series resonant frequency, respectively.

The parameters defined in Equations (1)–(5) are the equivalent electric parameters of a circular ring without considering silicon [40]. The resonant frequency considering the mass loading effect of silicon was computed as [40]

(6) $$f_{\mathrm{new}}=f_{s}\sqrt{\frac{1+\frac{E_{\mathrm{Si}}A_{\mathrm{Si}}}{E_{P}A_{P}}}{1+\frac{\rho_{\mathrm{Si}}A_{\mathrm{Si}}}{\rho_{P}A_{P}}}}$$

where *E* is the Young’s modulus, *A* is the cross-sectional area, and the subscripts Si and P point to the silicon and piezoelectric material. The parameter of piezoelectric material here was AlN and the calculated coefficient of mass loading was 0.77. The newly calculated series resonant frequency was 156.20 MHz after multiplying the coefficient of silicon.

**Figure 3:**
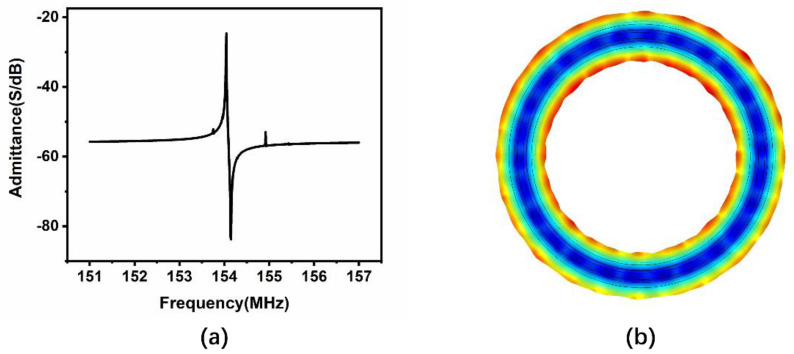
(**a**) Simulation result of the admittance versus frequency of the resonator, (**b**) vibration mode of resonator.

Finite element method (FEM) simulation was performed on the AlN ring type resonator. The Young’s modulus of silicon was 170 GPa, the density of silicon was 2329 $\mathrm{kg}/m^{3}$, and all parameters in simulation were the same as the parameters in the theoretical equations above. AC voltage was applied on top of the aluminum layer, and the bottom silicon layer was grounded. The admittance versus frequency result is illustrated in Figure 3a, and the series resonant frequency was 154 MHz, which is similar to the theoretical result. Figure 3b displays the vibration mode. For the piezoelectric coefficient *d*_31_ of AlN, the electric field in direction 3 (vertical) caused strain in direction 1 (horizontal), which showed the expanding/contracting vibration across the width direction of the ring.

## 3. Fabrication

**Figure 4:**
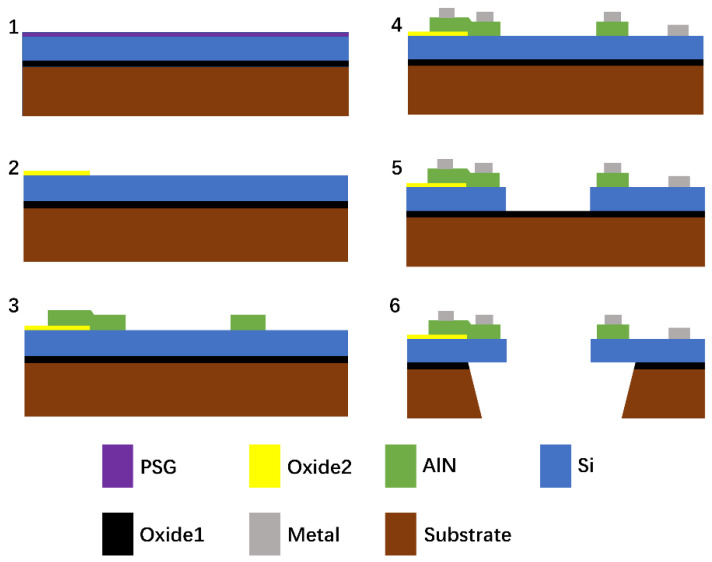
Schematic of the device fabrication process.

A contour mode resonator was successfully fabricated. The device fabrication process is shown in Figure 4, and the detail parameters are shown in Table 1. The fabrication process started with a SOI wafer. The first step was to deposit a phosphosilicate glass layer (PSG), and annealing was performed to drive the phosphorous dopant into the top silicon layer, which resulted in a n-type heavily doped silicon layer. The PSG layer was removed by 49% hydrofluoric (HF) solution. The second step was depositing a 200 nm of oxide, which isolated the pad metal to the ground; the oxide layer was patterned by photoresist, and etched by reactive ion etching (RIE). In the third step, a thin film of aluminum nitride with a thickness of 0.5 μm was deposited by reactive sputtering, patterned by photoresist and etched by wet etching (85% phosphoric acid). In the fourth step, the pad metal was deposited by electron beam evaporation through the liftoff process, containing 20 nm chromium and 1000 nm aluminum. Then, in the fifth step, deep reactive ion etching (DRIE) was applied to etch the silicon down to the oxide layer. Finally, the sixth step was the backside etching in order to release the resonator. RIE was used to remove the bottom oxide, and DRIE was applied to etch the substrate layer. The oxide layer underneath the silicon layer was etched using a buffered HF solution.

## 4. Results

**Figure 5:**
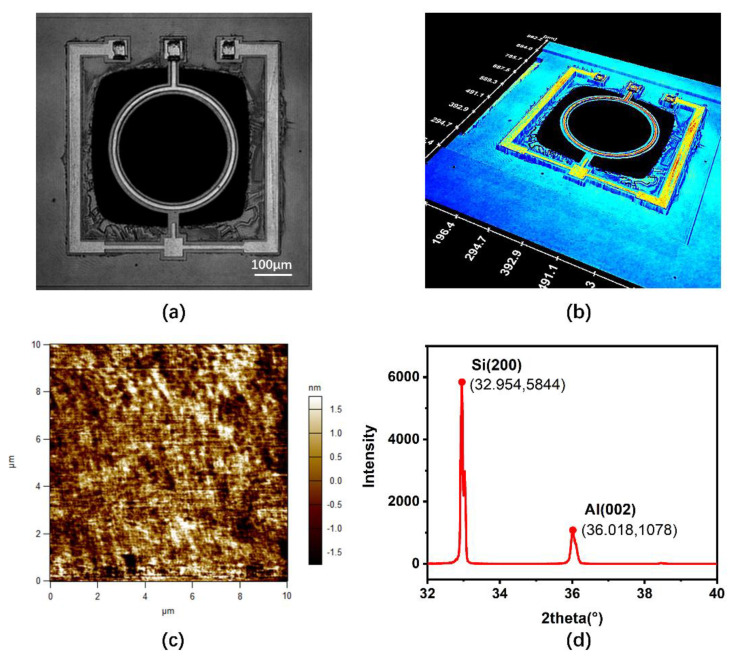
(**a**) 2D optical holographic image of the resonator, (**b**) 3D optical holographic image of the resonator, (**c**) Atomic force microscopy (AFM) image of AlN thin film, (**d**) X-ray diffraction patterns of AlN film.

An optical holographic microscope was applied to obtain the 2D and 3D images of the resonator, as shown in Figure 5a,b. Figure 5a shows that the geometric dimension was similar to that of the design model; the diameter of the ring was 312.3 μm, and the ring width of silicon, AlN, and aluminum was 29.54, 13.37, and 7.46 μm. The discrepancy between the measured AlN width and the designed AlN width was believed to be the result of the nonideal microfabrication process of wet etching. The backside of the SOI wafer was etched with sufficient area for the vibration of the resonator using DRIE technology. From the 3D image in Figure 5b, the measured thickness of silicon, AlN, and Al was shown to be 10.0 μm, 0.48 μm, and 1.06 μm, which means that the dimensions of the fabricated device is consistent with the design model.

Figure 5c illustrates the atomic force microscopy (AFM) image of AlN on top of the SOI substrate, with reactive sputtering technology applied to deposit the AlN layer. The AFM scan rate was at 2.44 Hz. The peak-to-valley (PV) value was measured as 8.68 nm and the root mean square (RMS) roughness was measured as 0.87 nm, while the lowest surface roughness in [41] was 2.3 nm, showing that the AlN film is very flat.

A high resolution X-ray Diffraction (XRD) diffractometer was applied to characterize the crystallographic structure of AlN. Figure 5d shows the XRD pattern of the AlN film on the silicon wafer. In the XRD pattern, (200) Si and (002) AlN were observed. The (002) peak of AlN film exhibited a preferred c-axis orientation [42]. The XRD spectra was obtained at a scanned angle of 2θ, varying from 32° to 40° in order to obtain a clearer result. Silicon was the base material, so the intensity was high, and the peak diffraction angle of Si (200) was 32.954°. The peak diffraction angle of AlN (002) was 36.018°, which is also close to the reported value by Kim et al. [42]. When calculated, the full width at half maximum (FWHM) value of AlN is 0.164°, which means the AlN film used was high quality *c*-axis AlN.

**Figure 6:**
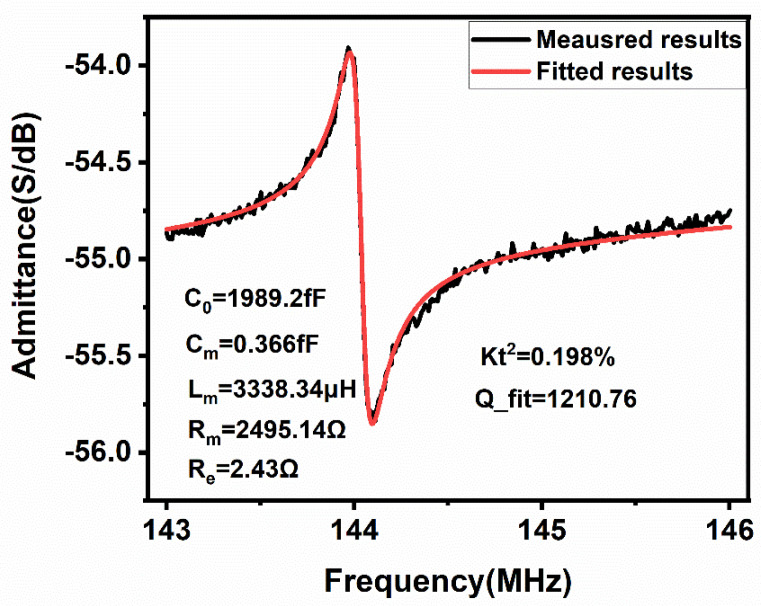
Results of the measured admittance versus frequency of the resonator.

We used a ZNB vector network analyzer (ZNB8, Rohde & Schwarz GmbH, Munich, Germany) and a MPI manual probe station (TS200,MPI Corporation, Suzhou, China) to measure the *S* parameter (return loss) versus frequency parameter, and the result was converted to admittance versus frequency (Y=1501−S1+S) shown in Figure 6. The black line is the simulation curve and the red line is the curve fitted by MATLAB (MATLAB R2019a, MathWorks, Natick, MA, USA). The series resonant frequency was determined from the measured frequency at minimum *S* parameter. The uncertainty of measurement was 0.01 dB in *S* parameter magnitude, and the accuracy of the measured frequency achieved a 10 kHz resolution. By comparing the experimental result with the simulation result, the resonant frequency was reduced by 6.5% from 154 MHz to 144.03 MHz, which is believed to be due to the mass loading of the top aluminum electrode. The quality factor *Q*_fit_ calculated in MATLAB is expressed as

(7) $$Q_{\mathrm{fit}}=2\pi f_{s}\frac{L_{m}}{R_{m}}$$

where *f*_s_ is the series resonant frequency, *L*_m_ is the motional inductance in Equation (2), and *R*_m_ is the motional resistance in Equation (4). The large motional resistance *R*_m_ of 2495.16 Ω and the low quality factor of 1210.76 in the experimental result is believed to be due to the mass loading effect of silicon and the anchor loss.

The electromechanical coupling coefficient $k_{t}^{2}$ calculated in MATLAB is expressed as

(8) $$k_{t}^{2}=\frac{f_{p}^{2}-f_{s}^{2}}{f_{p}^{2}}$$

where *f*_p_ is the parallel resonant frequency of the right vertex and *f*_s_ is the series resonant frequency of the left vertex on the curve and the calculated $k_{t}^{2}$ is 0.198%, which is believed to be due to the large thickness of silicon.

**Figure 7:**
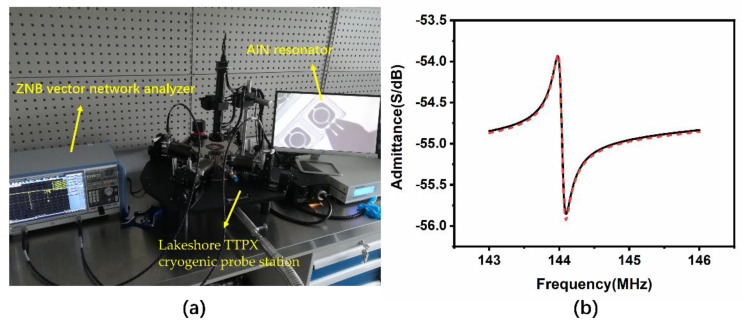
(**a**) ZNB Vector network analyzer and Lakeshore TTPX cryogenic probe station, (**b**) results of the measured admittance versus frequency of the resonator in atmospheric pressure (black line) and low pressure (red dotted line).

A ZNB vector network analyzer (ZNB8, Rohde & Schwarz GmbH, Munich, Germany) and Lakeshore TTPX cryogenic probe station (Lake Shore Cryotronics Inc, Columbus, OH, USA) were chosen to measure the TCF of the resonator, shown in Figure 7a. Cryogenic and high temperature characterization were performed within a wide temperature range, which is important in some special fields, such as aerospace industry. The quality factor versus temperature characteristics based on laterally-vibrating AlN resonator have been reported [43]; however, few studies have presented the cryogenic characteristics of contour mode AlN ring type resonator. Furthermore, few studies have reported the high temperature characteristics of AlN resonators with temperatures higher than 85 °C. We tested the performance of the AlN resonator versus temperature from 77 K to 400 K, covering both the low temperature and high temperature. The temperature variation must be tested at a low pressure; as a result, a measurement of the atmospheric pressure is done first to study whether the resonant frequency changes with pressure. We measured the admittance versus frequency characteristics of the resonators at both atmospheric pressure and a low pressure of 7.3 × 10^−5^ mBar in the Lakeshore TTPX cryogenic probe station, and the results are displayed in Figure 7b. The black curve is the atmospheric pressure result and the red dotted line is the low-pressure result. The series resonant frequencies were both 144.03 MHz, which means the resonant frequency was not sensitive to the pressure. For the vibration direction is the width direction, the air damping effect on contour mode resonator is slide film damping, which resulted in a parallel resistance [44]. So, the series resonant frequency is unaffected by the change in resistance and the quality factor is affected as Equation (7) (*R*_m_ is replaced by *R*_m_//*R*_air_). The quality factor increased from 1210.8 to 1291.4 when at the low pressure; lower pressure means smaller parallel resistance and higher quality factor. This phenomenon is also consistent with previous studies that showed that lower pressure results in a higher quality factor [45,46].

**Figure 8:**
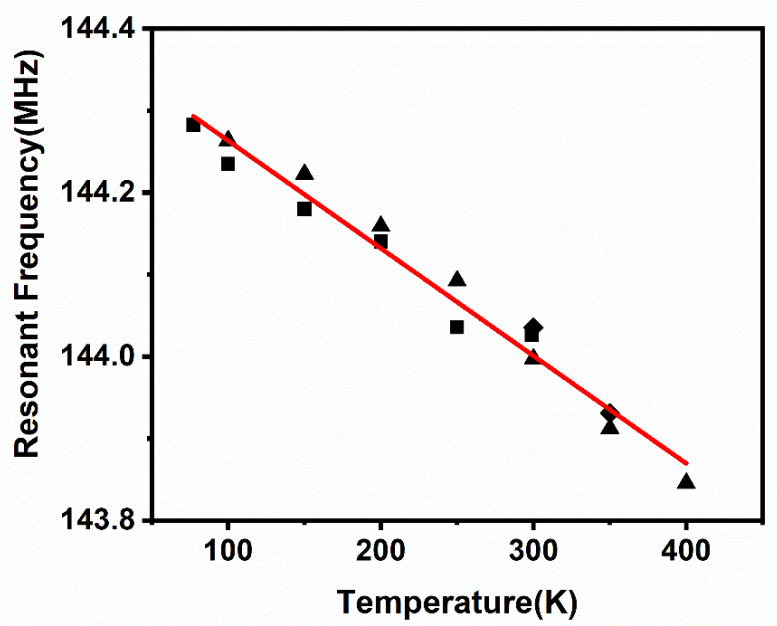
Results of the resonant frequency versus temperature of the resonator.

**Figure 9:**
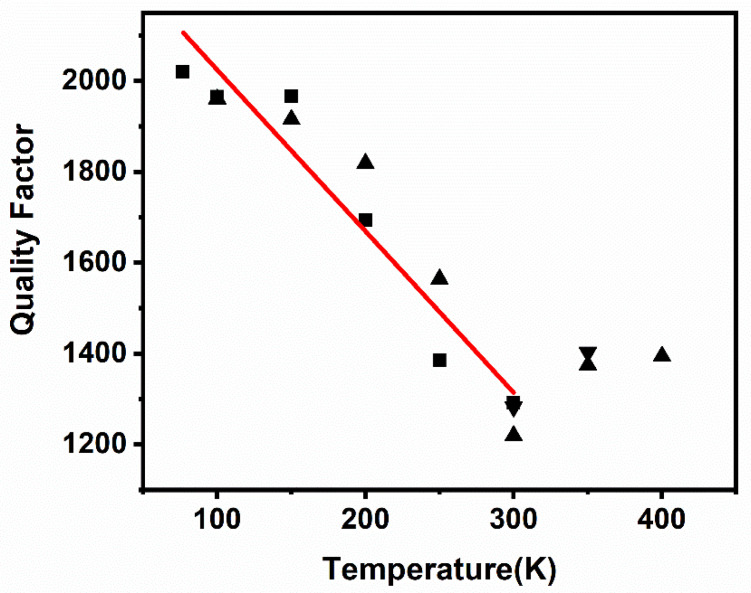
Results of the quality factor versus temperature of the resonator.

Cryogenic cooling was applied from room temperature to 77 K. Afterwards, the temperature was increased from 77 K to 400 K, and then cooled down to 300 K in order to characterize the temperature characteristics and the temperature hysteresis effect on the AlN resonator. The experimental results are shown in Figure 8 and Figure 9, respectively. The experimental results from room temperature (300 K) to 77 K are denoted by the square data points, the results from 77 K to 400 K are denoted by the triangle data points, and the results of the temperature drop from 400 K to 300 K are denoted by the diamond data points. The resonant frequency increased by 0.18% from 144.03 MHz at the temperature of 300 K to 144.28 MHz at the temperature of 77 K, fitting the negative temperature coefficient of the theoretical TCF. The results with the fitted line reveal that the resonant frequency varied linearly with temperature. The linear equation to fit the result is expressed as

(9) f=aT+b

where *a* is −0.00131 MHz/K and *b* is 144.39 MHz. From this equation, we can interpolate that the resonant frequency close to 0 K is 144.39 MHz, and it changes 0.25% from 300 K. Compared to the TCF of the contour mode AlN resonator, which is −25 ppm/°C [40], the ring type resonator in this paper has a lower TCF of −9.1 ppm/°C.

Because of the silicon bottom electrode of this AlN resonator, the theoretical TCF analysis was performed for this composite structure, and the resonant frequency is calculated as [47]

(10) $$f=\frac{1}{2\pi }\sqrt{\frac{\sum_{n=1}^{N}{\left(EA\right)}_{n}}{mR}}$$

(11) $$f^{2}=\frac{m_{\mathrm{Si}}}{m}f_{\mathrm{Si}}{}^{2}+\frac{m_{\mathrm{AlN}}}{m}f_{\mathrm{AlN}}{}^{2}$$

where *E* is the Young’s modulus, *A* is the cross-section area, *m* is the total mass of both Si and AlN, and *R* is the radius of ring. The effect of aluminum is neglected in Equation (11). For the contour mode, the TCF expression can be simplified as [47]

(12) $$TCF=\frac{{\left(TCF\right)}_{\mathrm{AlN}}+r{\left(TCF\right)}_{\mathrm{Si}}}{1+r}$$

(13) $$r=\frac{E_{\mathrm{Si}}A_{\mathrm{Si}}}{E_{\mathrm{AlN}}A_{\mathrm{AlN}}}$$

where $E_{\mathrm{Si}}$ is 170 GPa, $E_{\mathrm{AlN}}$ is 99 GPa, $A_{\mathrm{Si}}$ is 295.4 μm^2^, and $A_{\mathrm{AlN}}$ is 6.42 μm^2^. The calculated value *r* is 79.01, which means the TCF of this structure is mainly determined by the TCF of silicon. The TCF of AlN is −25 ppm/°C [24], and the TCF of heavily doped silicon is −8 ppm/°C [33]. Therefore, the calculated TCF of the resonator is −8.21 ppm/°C. As a result, the experimental TCF was consistent with the theoretical result. The temperature hysteresis effect was also negligible, as shown in Figure 8; the resonant frequency was shifted by 0.03 MHz at 100 K, 0.04 MHz at 150 K, 0.02 MHz at 200 K, 0.06 MHz at 250 K, 0.03 MHz at 300 K, and 0.02 MHz at 350 K, which exemplifies the temperature stability in this device.

The quality factor for the MEMS resonator can be influenced by many parameters, such as air damping (*Q*_air_), which can be neglected for the low pressure in this measurement, thermoelastic dissipation (*Q*_TED_), energy loss from anchor (*Q*_anchor_), and other situations (*Q*_others_) shown in Equation (14).

(14) $$\frac{1}{Q_{\mathrm{All}}}=\frac{1}{Q_{\mathrm{TED}}}+\frac{1}{Q_{\mathrm{anchor}}}+\frac{1}{Q_{\mathrm{others}}}$$

The anchor loss has a negligible temperature dependence, and the temperature dependence of *Q*_TED_ can be expressed as [46]

(15) $$Q_{\mathrm{TED}}=Q_{\mathrm{TED},\mathrm{freq}}Q_{\mathrm{TED},\mathrm{mat}}=\frac{f_{M}^{2}+f_{T}^{2}}{f_{M}f_{T}}Q_{\mathrm{TED},\mathrm{mat}}$$

where *Q*_TED,freq_ is the frequency term, *Q*_TED,mat_ is the material term, *f*_M_ is the mechanical mode frequency, and *f*_T_ is the thermal mode frequency. At a given mechanical mode frequency, *f*_T_ decreased as the temperature increased, and the *Q*_TED_ value reached the minimum when *f*_T_ was the same as *f*_M_ [46].

The measured result of the quality factor versus temperature characteristics is displayed in Figure 9. Based on the measurement result, we estimated that *f*_T_ was close to *f*_M_, around 300 K. As a result, the quality factor decreased with the increase in temperature when the temperature was lower than 300 K, while the quality factor increased with the increase of temperature when the temperature was higher than 300 K. Based on Figure 9, a linear relationship between quality factor and temperature was found when the temperature is below 300 K. The quality factor versus temperature characteristics from 77 K to 300 K can be linearly fitted by Equation (16)

(16) Q=cT+d

where *T* is the temperature, *c* is −3.55/K, and *d* is 2379.45. The quality factor increased by 56.43% from 1291.4 at the temperature of 300 K, to 2020.2 at the temperature of 77 K in measurement. The quality factor in a linearly fitted curve changed 0.27% per degree of temperature change, which was lower than that of 1% per degree of temperature change shown by Kim et al. [46].

## 5. Conclusions

AlN resonators are attractive due to their high acoustic wave velocity and excellent thermal conductivity. However, the TCF of AlN is −25 ppm/°C, which is too high for filters and sensing applications. Previous studies have reported that active and passive temperature compensation technologies may reduce the TCF. However, active compensation techniques add circuit complexity/power consumption, and passive compensation techniques increase the fabrication complexity. In this study, a contour mode ring type AlN resonator with n-type heavily doped silicon as the bottom electrode is presented with a resonant frequency of 144.03 MHz and an electromechanical coupling coefficient of 0.198%. The resonator was fabricated on a SOI substrate to simplify the fabrication process. The thickness ratio of the silicon bottom electrode to AlN layer was 20:1, which means that the TCF is mainly determined by silicon. Low TCF heavily doped silicon was implemented to reduce the TCF of the resonator. The cryogenic and high temperature experiment showed that resonant frequency and quality factor varied linearly with temperature. The measured TCF of the resonator was −9.1 ppm/°C, which means that the heavily doped silicon reduced the TCF of the resonator. Low temperature hysteresis was observed, which shows that the ring type contour mode AlN resonator had good temperature stability. The drawback of this resonator is the low quality factor and high motional resistance due to the heavily doped silicon. In future, single support or notched support [40] will be studied to replace the double support used in this design, which will reduce the anchor loss effect in order to reduce the motional resistance and increase the quality factor.

## Figures and Tables

**Table 1:** Parameters of the fabrication process.

Parameters	Value in μm
Thickness of top silicon layer	10
Thickness of AlN layer	0.5
Width of silicon ring	30
Width of AlN ring	16.6
Width of Cr/Al ring	8
Radius of Si/AlN/Cr/Al ring	160
